# Physical activity levels and HINT-8 health-related quality of life in Korean adults with diabetes: analysis of KNHANES 2019–2021

**DOI:** 10.1007/s11136-026-04265-1

**Published:** 2026-05-03

**Authors:** Sung Hoon Jeong, You-Jung Choi, Gain Shin, Ja-Ho Leigh

**Affiliations:** 1 National Traffic Injury Rehabilitation Research Institute, National Traffic Injury Rehabilitation Hospital, Yangpyeong, 12564 Republic of Korea; 2 Department of Health Policy & Management, Inje University, Gimhae, 50834 Republic of Korea; 3 Institute of Health Services Research, Yonsei University, Seoul, Republic of Korea; 4 Department of Nursing, Seojeong University, Yangju-Si, 11429 Republic of Korea; 5 Department of Rehabilitation Medicine, Seoul National University Hospital, 101 Daehak-Ro, Jongno-Gu, Seoul, 03080 Republic of Korea; 6 Institute of Health Policy and Management, Medical Research Center, Seoul National University, Seoul, Korea

**Keywords:** Health-related quality of life, Health-related Quality of Life Instrument with 8 Items (HINT-8), Physical activity, Diabetes mellitus, Cross-sectional study, Korea National Health and Nutrition Examination Survey (KNHANES)

## Abstract

**Purpose:**

Physical activity is a core component of diabetes management. However, evidence linking physical activity levels to health-related quality of life (HR-QOL) in adults with diabetes remains limited, particularly when measured using the Health-related Quality of Life Instrument with 8 Items (HINT-8). This study examined the association between physical activity level and HR-QOL and explore subgroup patterns among Korean adults with diabetes.

**Methods:**

We analyzed nationally representative data from the 8th Korea National Health and Nutrition Examination Survey (2019–2021). Adults with diabetes were identified using fasting plasma glucose, glycated hemoglobin, or prior diagnosis/medication use. HR-QOL was assessed using HINT-8, a preference-based instrument developed to reflect the health preferences of the general Korean population. Physical activity levels were classified as low, moderate, or high using the Global Physical Activity Questionnaire. Associations were examined using survey-weighted linear regression.

**Results:**

Among 1,590 adults with diabetes, 59.3% reported low physical activity. Compared with the low-activity group, high physical activity was significantly associated with higher HINT-8 scores (β = 0.024; 95% CI 0.008–0.039). Although the moderate activity group showed a positive trend (β = 0.010; 95% CI − 0.004 to 0.023), it did not reach statistical significance.

**Conclusion:**

High physical activity levels may be associated with better perceived health status among Korean adults with diabetes, highlighting the importance of supporting adults with diabetes to achieve higher overall physical activity levels.

**Supplementary Information:**

The online version contains supplementary material available at 10.1007/s11136-026-04265-1.

## Plain English summary

Physical activity is an important part of diabetes care, but less is known about how the overall physical activity level is related to quality of life in Korean adults with diabetes. This study analyzed whether higher levels of activity, combining intensity, frequency, and duration, were associated with quality of life. It also explored whether different patterns were seen across education, job type, and the presence of other health conditions, such as high blood pressure or high cholesterol. After accounting for sociodemographic and health factors, people who engaged in a high level of physical activity reported a better quality of life than those who had low activity levels. While moderate activity showed a slightly positive trend, it was not statistically significant, suggesting that substantial activity may be required for noticeable benefits. Because most participants reported low activity, our findings suggest a gap between the recommended and actual activity levels. Overall, the results suggest that supporting Korean adults with diabetes in achieving higher overall physical activity levels may help improve their perceived well-being.

## Background

Diabetes mellitus is a major and rapidly growing global health challenge. The International Diabetes Federation reported that approximately 537 million adults aged 20–79 years (10.5% of the adult population) were living with diabetes in 2021, a figure projected to increase to nearly 783.2 million (12.2%) by 2045 [1]. In South Korea, an estimated 15–16% of adults aged ≥ 30 years, equivalent to approximately 5–6 million people, have diabetes, and nearly one-third of those aged ≥ 65 years are affected. Despite this substantial burden, fewer than one-third of patients achieve integrated management targets for glycated hemoglobin (HbA1c), blood pressure, and low-density lipoprotein cholesterol [2]. Of particular concern is the rising prevalence of diabetes among younger adults aged 30–49 years, who have lower levels of disease awareness, treatment uptake, and glycemic control, thereby foreshadowing a substantial future burden of cardiovascular and renal complications. Because diabetes requires lifelong self-management and is strongly associated with vascular complications and premature mortality, health-related quality of life (HR-QoL) has emerged as a critical patient-centered outcome and independent prognostic indicator [3, 4]. Lower HR-QoL scores have been shown to independently predict all-cause and cardiovascular mortality in individuals with diabetes [5, 6], underscoring the need to prioritize HR-QoL as a core objective of comprehensive diabetes care.

Regular physical activity serves as a cornerstone of diabetes management [7]. The World Health Organization (WHO) recommends that adults, including those with chronic conditions, accumulate 150–300 min per week of moderate-level aerobic activity or 75–150 min of vigorous aerobic activity, in addition to muscle-strengthening exercises for at least 2 days per week [8]. Pooled analysis of prospective cohort studies has demonstrated an inverse association between physical activity and incident diabetes, exhibiting a clear dose–response gradient [9]. Among adults with established diabetes, exercise improves glycemic control, cardiorespiratory fitness, and cardiometabolic risk factors. Moreover, structured exercise programs have been shown to yield small-to-moderate improvements in HR-QoL [3, 7]. Observational studies in adults with diabetes have similarly reported that individuals who are more physically active, or those who meet aerobic activity recommendations, tend to report higher HR-QoL than their inactive counterparts [10–12]. Most prior studies have emphasized adherence to activity guidelines over graded physical activity levels, and few have considered domain-specific activity (work-related, transport-related, and leisure-time) when examining HR-QoL [10–12]. This gap has raised questions about whether the health benefits of physical activity accrue uniformly across different activity contexts and socio-economic groups, particularly considering the “physical activity paradox,” whereby high levels of occupational physical activity may not confer the same benefits as leisure-time physical activity [13, 14].

HR-QoL has frequently been assessed using generic instruments such as the EuroQol-5 Dimension (EQ-5D), which reflects basic functional health status [15]. The EQ-5D may overlook subtle yet clinically relevant symptoms common in diabetes, such as fatigue, sleep disturbance, and cognitive burden, which contribute to the invisible symptoms and distress burden of the disease [16]. To address concerns related to cultural relevance and measurement sensitivity, the Health-related Quality of Life Instrument with 8 Items (HINT-8) was developed based on health-state valuations derived from the general population of Korea and has been incorporated into the KNHANES as a biennial rotating item since 2019 [17]. The HINT-8 is a brief, preference-based measure encompassing physical, social, mental, and positive health domains; importantly, it includes items on vitality, memory, and sleep, which may be particularly sensitive to the diffuse symptom burden and diabetes-related distress experienced by adults with diabetes [17–19]. However, whether GPAQ-based physical activity levels are associated with HR-QoL, as assessed using this more sensitive utility-based measure, in adults with diabetes remains unclear. Therefore, we sought to examine the association between physical activity levels and HR-QoL among Korean adults with diabetes mellitus. The secondary objective of this study was to investigate whether these associations differed according to socio-economic status, occupational category, and cardiometabolic multimorbidity.

## Methods

### Data and study population

This study utilized data from the KNHANES-VIII (2019–2021), a nationwide, cross-sectional survey conducted by the Korea Disease Control and Prevention Agency (KDCA). KNHANES is a nationwide population-based cross-sectional survey that assesses the health and nutritional status of the Korean population [20]. The KNHANES employs a stratified, multistage, clustered sampling methodology to select participants, thereby providing reliable and nationally representative data on the Korean population and serving as a key resource for the development and evaluation of national health policies and programs. Written informed consent was obtained by the KDCA from all respondents who participated in the KNHANES, and all data analyzed in this study were fully anonymized [20]. This study was conducted in accordance with the Declaration of Helsinki and was exempted from review by the Institutional Review Board (IRB) of Seoul National University Hospital (IRB No. SNUH 2025-12-002) as it utilized de-identified secondary data.

**Fig. 1:**
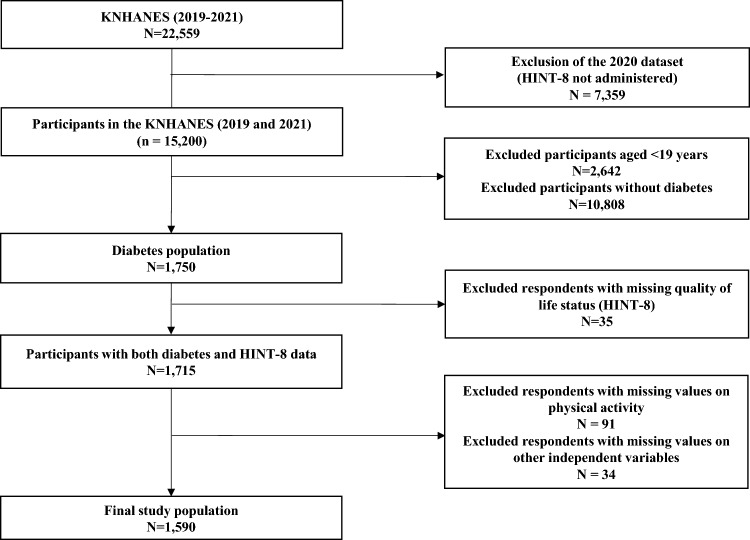
Flowchart for participant inclusion. KNHANES, Korea National Health and Nutrition Examination Survey; HINT-8, 8-item Health-Related Quality of Life

The initial KNHANES-VIII dataset comprised 22,559 individuals. However, because the HINT-8 was administered as a biennial rotating survey item, it was not conducted in 2020 [21]. Consequently, 7,359 participants from the 2020 survey cycle were excluded from the analysis. The combined KNHANES-VIII dataset from the 2019 and 2021 survey waves (in which HINT-8 data were available) comprised a total of 15,200 individuals. First, participants younger than 19 years were excluded (n = 2,642). Second, diabetes was defined according to the following criteria: (i) fasting plasma glucose (FPG) ≥ 126 mg/dL or HbA1c ≥ 6.5%; or (ii) a prior diagnosis of diabetes by a physician or current use of antidiabetic medication [2, 22]. Individuals who did not meet these criteria were excluded (n = 10,808). Third, 35 individuals with incomplete or missing HINT-8 data were excluded, leaving 1,715 participants. Subsequently, 91 individuals with missing physical activity data were excluded. Finally, 34 participants with missing data for the other independent variables included in the analysis were excluded. Ultimately, the final study sample comprised 1,590 participants. A flow chart illustrating the participant selection process is shown in Fig. 1.

### Variables

HR-QoL, the primary dependent variable in this study, was assessed using the HINT-8 instrument [23]. Detailed information regarding the development and validation of the HINT-8 is available in prior publications and on the National Health Insurance website. The HINT-8 was specifically developed to reflect the health preferences of the general population in Korea and consists of eight items covering the physical (climbing stairs, pain, and energy), social (working), mental (depression, memory, and sleep), and positive health domains. Each item is rated on a four-level response scale, where level 1 indicates no problem and level 4 indicates a very severe problem. By combining the response levels across all items, 65,536 unique health states can be defined. Using the formula developed for the entire population of Korea, the health utility index can be calculated for each HINT-8 health state, ranging from an index value of 1 for perfect health (11,111,111) to 0.13 for the worst health state (44,444,444) [17, 24]. In this study, the HINT-8 utility scores were calculated according to the scoring algorithm reported in previous research [17, 25].

Physical activity level, the primary independent variable in this study, was measured using the Global Physical Activity Questionnaire (GPAQ). The GPAQ has been translated into Korean, and its validity and reliability have been previously established [26, 27]. The GPAQ assesses physical activity across three domains: work-related, transport-related, and leisure-time activity. In the work-related and leisure-time domains, the frequency and duration of activities performed at two intensity levels, vigorous and moderate, were assessed. In the transport-related domain, the frequency and duration of walking and cycling for travel were evaluated. Total physical activity was estimated by summing the metabolic equivalent task (MET) minutes per week spent for moderate and vigorous activities, including walking and cycling (moderate-intensity MET value = 4.0, vigorous-intensity MET value = 8.0, walking and cycling MET value = 4.0) [28]. These MET-minutes/week represent a composite measure that incorporates the intensity, frequency, and total volume of physical activity performed during a typical week. Participants were then classified into three physical activity level categories, low, moderate, and high, according to GPAQ guidelines based on combinations of weekly frequency, duration, and total MET-minutes/week (Supplementary material) [29, 30].

The independent variables considered as potential confounders included sociodemographic characteristics, health-related factors, and the survey year. Socio-economic characteristics included age (19–29, 30–39, 40–49, 50–59, 60–69, and ≥ 70 years), sex (male or female), marital status (married, single, widowed, separated, or divorced), education level (middle school or lower, high school, or college or higher), area of residence (urban or rural), household income (low, mid-low, mid-high, or high), and occupation (white-collar, pink-collar, blue-collar, or economically inactive [termed ‘inoccupation’ in the KNHANES classification]). Occupations were classified according to the Korean version of the Standard Classification of Occupations, which is based on the International Standard Classification of Occupations established by the International Labor Organization. Therefore, white-collar occupations refer to office workers, pink-collar occupations include sales and service jobs, and blue-collar occupations include agriculture, forestry, fishery, and military service occupations, following the Korean Standard Classification of Occupations. Health-related characteristics included body mass index (BMI), chronic disease status (yes or no), drinking status (yes or no), and smoking status (yes or no). BMI was classified according to the practice guidelines of the Korean Society of Obesity [31]: < 23.0 kg/m^2^ as underweight or normal weight, 23.0–24.9 kg/m^2^ as overweight, and ≥ 25.0 kg/m^2^ as obesity. In addition, cardiometabolic multimorbidity was defined as the co-occurrence of diabetes with at least one additional physician-diagnosed condition: hypertension or dyslipidemia [32].

### Statistical analysis

Survey-weighted simple linear regression was performed to evaluate the relationships between physical activity level and HR-QoL in patients with diabetes, as well as associations with sociodemographic variables, health-related variables, and survey year. For categorical variables with more than two levels, overall differences were assessed using F-tests derived from the survey-weighted linear regression models. Survey-weighted multiple linear regression analysis was then conducted to examine the association between physical activity level and HR-QoL after adjusting for potential confounding variables, including sociodemographic characteristics, health-related factors, and survey year. Further, subgroup analyses were conducted using survey-weighted multiple linear regression models stratified by sex, education level, occupational category, and chronic disease status to investigate the associations between physical activity level and HR-QoL.

To account for the complex sampling design of the KNHANES, all statistical analyses were performed using the PROC SURVEYREG procedure, incorporating stratification variables, clustering variables, and sampling weights. Because data from two survey years (2019 and 2021) were combined, the sampling weights were divided by two in accordance with the KNHANES analytical guidelines. Although the HINT-8 index exhibits a skewed distribution, survey-weighted linear regression models were utilized, as they provide robust standard errors and allow for the direct interpretation of absolute differences in utility scores. The estimated regression coefficients (β) represent the absolute mean difference in HINT-8 scores compared with the reference group. All statistical analyses were performed using SAS software, version 9.4 (SAS Institute, Inc.), and statistical significance was defined as a *P*-value of < 0.05.

### Ethical considerations

This study was exempted from review by the Institutional Review Board of Seoul National University Hospital (IRB No. SNUH 2025-12-002) because it utilized publicly available, de-identified secondary data. Written informed consent was originally obtained from all participants by the KDCA during the primary KNHANES data collection. For this secondary analysis, the requirement for additional informed consent was waived by the IRB.

## Results

The general characteristics of the study population are presented in Table 1. Of the 1,590 participants, 825 (57.4%) were men and 765 (42.6%) were women. Among the participants, 959 (59.3%) were in the low physical activity group, 414 (26.7%) in the moderate group, and 217 (14.0%) in the high group. Using survey-weighted simple linear regression, we confirmed that the differences in mean HR-QoL scores across physical activity levels were statistically significant based on F-tests (*P* < .0001). Furthermore, all socio-economic and health-related characteristics were statistically significantly associated with HR-QoL, with the exception of region, BMI, smoking status, and survey year.

**Table 1:** General characteristics of the study population

Variables	Total(n = 1,590)				
	Participants		Hint-8		
	N	Weighted %	Mean	SE	*P* value
Physical activity					< .0001
Low	959	59.3	0.774	0.004	
Moderate	414	26.7	0.794	0.006	
High	217	14.0	0.815	0.007	
Sex					< .0001
Male	825	57.4	0.805	0.004	
Female	765	42.6	0.759	0.004	
Age					< .0001
19–29	12	1.5	0.824	0.024	
30–39	45	4.2	0.830	0.014	
40–49	168	14.4	0.811	0.007	
50–59	281	24.0	0.805	0.006	
60–69	507	29.2	0.784	0.006	
≥ 70	577	26.7	0.746	0.006	
Education level					< .0001
Middle school or under	816	41.7	0.754	0.005	
High school	459	33.6	0.803	0.005	
College or over	315	24.8	0.814	0.005	
Region					0.5621
Urban area	1148	77.5	0.786	0.003	
Rural area	442	22.5	0.782	0.006	
Household income					< .0001
Low	500	24.8	0.737	0.007	
Mid-low	439	27.2	0.784	0.006	
Mid-high	349	24.9	0.811	0.005	
High	302	23.1	0.811	0.006	
Occupational categories^a^					< .0001
White	204	16.5	0.825	0.005	
Pink	145	10.2	0.798	0.008	
Blue	496	31.0	0.803	0.005	
Inoccupation	745	42.2	0.753	0.005	
Marital Status					< .0001
Married	1144	74.0	0.798	0.003	
Single, widow, divorced, separated	446	26.0	0.748	0.007	
BMI					0.3456
Under/Normal	387	23.5	0.778	0.007	
Over	357	20.7	0.784	0.006	
Obesity	846	55.8	0.789	0.004	
Chronic diseases^d^					< .0001
No	441	31.1	0.809	0.005	
Yes	1149	68.9	0.774	0.004	
Drinking status					< .0001
No	636	35.8	0.763	0.005	
Yes	954	64.2	0.798	0.004	
Smoking status					0.7054
No	1303	78.7	0.785	0.003	
Yes	287	21.3	0.788	0.008	
Year					0.1729
2019	786	47.2	0.790	0.005	
2021	804	52.8	0.781	0.004	
Total	1,590	100.0	0.8	0.1	

Data are presented as unweighted numbers (N) and weighted percentages (%)

HINT-8 scores are presented as weighted means and standard error (SE)

P-values were calculated using F-tests from survey-weighted simple linear regression models to account for the complex sampling design of the KNHANES

^a^Occupational categories are classified into white-collar, pink-collar, and blue-collar groups based on the International Standard Classification of Occupations codes. The “inoccupation” group includes homemakers

^b^Body mass index (BMI) categories are defined based on the 2018 Clinical Practice Guidelines for Overweight and Obesity in Korea: underweight or normal (< 23.0 kg/m^2^), overweight (23.0–24.9 kg/m^2^), and obese (≥ 25.0 kg/m^2^)

^c^Cardiometabolic multimorbidity: includes diagnosed diseases such as hypertension and dyslipidemia

*HINT-8* Health-related Quality of Life Instrument with 8 Items

The association between physical activity levels and HR-QoL after adjusting for all control variables is summarized in Table 2. Compared to participants in the low physical activity group, the HINT-8 score was 0.024 points higher (95% confidence interval [CI] 0.008–0.039; *P* = 0.0035) among those in the high physical activity group, which was statistically significant. Although the moderate physical activity group showed a positive trend (β = 0.010), the difference was not statistically significant (95% CI − 0.004 to 0.023; *P* = 0.1615).

**Table 2:** Results of the analysis of factors associated with the HINT-8 index

Variables	HINT-8				
	Estimate (β)	95% CI			*P* value
Physical activity					
Low	Ref				
Moderate	0.010	(− 0.004	to	0.023)	0.1615
High	0.024	(0.008	to	0.039)	0.0035
Sex					
Male	Ref				
Female	− 0.024	(− 0.036	to	− 0.011)	0.0002
Age					
19–29	Ref				
30–39	− 0.018	(− 0.081	to	0.044)	0.5613
40–49	− 0.042	(− 0.098	to	0.015)	0.1497
50–59	− 0.036	(− 0.093	to	0.021)	0.2142
60–69	− 0.041	(− 0.098	to	0.017)	0.1628
≥ 70	− 0.049	(− 0.106	to	0.008)	0.0904
Education level					
Middle school or under	Ref				
High school	0.022	(0.007	to	0.037)	0.0038
College or over	0.021	(0.004	to	0.039)	0.0177
Region					
Urban area	Ref				
Rural area	0.002	(− 0.010	to	0.015)	0.7168
Household income					
Low	Ref				
Mid-low	0.022	(0.003	to	0.040)	0.0228
Mid-high	0.036	(0.017	to	0.056)	0.0003
High	0.029	(0.010	to	0.049)	0.0035
Occupational categories^a^					
White	0.024	(0.006	to	0.043)	0.0110
Pink	0.017	(− 0.003	to	0.036)	0.0924
Blue	0.029	(0.014	to	0.044)	0.0001
Inoccupation	Ref				
Marital Status					
Married	Ref				
Single, widow, divorced, separated	− 0.026	(− 0.040	to	− 0.012)	0.0004
BMI					
Under/Normal	Ref				
Over	0.005	(− 0.012	to	0.022)	0.5561
Obesity	0.002	(− 0.013	to	0.017)	0.8177
Chronic diseases^d^					
No	Ref				
Yes	− 0.019	(− 0.032	to	− 0.007)	0.0018
Drinking status					
No	Ref				
Yes	0.002	(− 0.011	to	0.015)	0.7470
Smoking status					
No	Ref				
Yes	− 0.021	(− 0.036	to	− 0.006)	0.0055
Year					
2019	Ref				
2021	− 0.01	(− 0.020	to	0.002)	0.1048

^a^Occupational categories are classified into white-collar, pink-collar, and blue-collar groups based on the International Standard Classification of Occupations codes. The “inoccupation” group includes homemakers

^b^Body mass index (BMI) categories were defined based on the 2018 Clinical Practice Guidelines for Overweight and Obesity in Korea: underweight or normal (< 23.0 kg/m^2^), overweight (23.0–24.9 kg/m^2^), and obese (≥ 25.0 kg/m^2^)

^c^Cardiometabolic multimorbidity: includes diagnosed diseases such as hypertension and dyslipidemia

*HINT-8* Health-related Quality of Life Instrument with 8 Items

Table 3 presents the results of the subgroup analyses stratified by independent variables, along with the formal interaction p-values for each stratification variable. Formal interaction tests between physical activity level and each stratification variable revealed no statistically significant effect modification (PA × sex: P = 0.331; PA × education: P = 0.940; PA × occupation: P = 0.562; PA × chronic disease: P = 0.298; Table 3). Within each stratum, a generally consistent positive trend was observed across physical activity levels. Among the sex subgroups, high physical activity was significantly associated with higher HINT-8 scores in both men (β = 0.021, 95% CI 0.002–0.041, *P* = 0.029) and women (β = 0.027, 95% CI 0.000–0.054, *P* = 0.048). Regarding educational level, a significant association was observed only in the college or higher subgroup (high activity: β = 0.034, 95% CI 0.005–0.062, *P* = 0.020). Among the occupational categories, significant associations with high physical activity were found in the white-collar (β = 0.035, 95% CI 0.004–0.066, *P* = 0.027) and inoccupation groups (β = 0.042, 95% CI 0.010–0.074, *P* = 0.010), whereas no significant associations were observed in the pink-collar or blue-collar groups. Among participants with chronic diseases, high physical activity was significantly associated with better HR-QoL (β = 0.033, 95% CI 0.012–0.054, *P* = 0.002), whereas no significant association was found in those without chronic diseases.

**Table 3:** Results of the subgroup analysis of physical activity with the HINT-8 index

Variables	HINT-8 Index											
	Physical activity											
	Low	Moderate					High					
	Estimate (β)	Estimate (β)	95% CI			*P* value	Estimate (β)	95% CI			*P* value	P for interaction
Sex												0.3308
Male	Ref	0.003	− 0.015	to	0.021	0.775	0.021	0.002	to	0.041	0.029	
Female	Ref	0.021	0.001	to	0.041	0.036	0.027	0.001	to	0.054	0.048	
Education level												0.9396
Middle school or under	Ref	0.011	− 0.011	to	0.033	0.325	0.021	− 0.008	to	0.050	0.154	
High school	Ref	0.003	− 0.023	to	0.028	0.846	0.017	− 0.009	to	0.043	0.195	
College or over	Ref	0.009	− 0.013	to	0.031	0.413	0.034	0.005	to	0.062	0.020	
Occupational categories^a^												0.5621
White	Ref	0.018	− 0.005		0.042	0.122	0.035	0.004	to	0.066	0.027	
Pink	Ref	0.007	− 0.023	to	0.036	0.651	− 0.008	− 0.043	to	0.027	0.651	
Blue	Ref	0.004	− 0.016	to	0.024	0.695	0.003	− 0.018	to	0.025	0.769	
Inoccupation	Ref	0.011	− 0.015	to	0.037	0.403	0.042	0.010	to	0.074	0.010	
Chronic diseases^b^												0.2981
No	Ref	− 0.001	− 0.028	to	0.027	0.969	0.010	− 0.015	to	0.035	0.431	
Yes	Ref	0.014	− 0.002	to	0.030	0.078	0.033	0.012	to	0.054	0.002	

P for interaction was derived from the F-test of the physical activity level × subgroup variable interaction term in each survey-weighted linear regression model

^*^Adjusted for all other covariates

^a^Occupational categories are classified into white-collar, pink-collar, and blue-collar groups based on the International Standard Classification of Occupations codes. The “inoccupation” group includes homemakers

^b^Cardiometabolic multimorbidity: includes diagnosed diseases such as hypertension and dyslipidemia

*HINT-8* Health-related Quality of Life Instrument with 8 Items

## Discussion

Using nationally representative data from KNHANES-VIII, we found that a high physical activity level was significantly associated with higher HR-QoL, measured by the HINT-8 utility index, among Korean adults with diabetes. Compared to the low-activity group, individuals in the high physical activity level group showed a significantly higher HINT-8 score. While the moderate physical activity level group also exhibited a positive trend, this association did not reach statistical significance, suggesting that a higher threshold of physical activity may be required to achieve statistically significant gains in utility-based QoL. Subgroup analyses further indicated that this significant positive association with high activity levels was consistent across sexes and was particularly pronounced among those with higher education, white-collar or inoccupation status, and cardiometabolic multimorbidity. These findings suggest a significant association between high physical activity levels and perceived health status in adults with diabetes, underscoring the role of high physical activity in promoting well-being beyond conventional biomedical outcomes. Our results indicate that achieving a high physical activity level may be necessary to yield statistically significant gains in HR-QoL, as these levels represent a total activity dose that integrates intensity, frequency, and duration.

Despite these beneficial associations, only 26.7% and 14.0% of participants were in the moderate and high physical activity groups, respectively, whereas 59.3% remained at a low physical activity level. These results indicate that more than half of Korean adults with diabetes may not meet the minimum level of physical activity recommended by the WHO [8]. This finding is consistent with that reported in previous Korean surveillance data demonstrating similarly low adherence to physical activity guidelines among individuals with diabetes [33]. Both the Korean Diabetes Association and the American Diabetes Association recommend at least 150 min of moderate-intensity or higher aerobic activity per week, in combination with resistance training two to three times per week [31, 34]. However, our findings suggest a significant gap between these clinical guidelines and real-world practice. Given that a statistically significant association with better HR-QoL was primarily observed in the high physical activity group, this low adherence implies that many adults with diabetes may not be reaching the threshold of activity required for significant HR-QoL gains. Since higher physical activity levels, particularly those involving vigorous activity, are known to elicit greater improvements in insulin sensitivity, endothelial function, and anti-inflammatory responses, this gap suggests that many patients are missing out on the meaningful benefits that high-level activity confers on both metabolic regulation and perceived well-being [35]. Therefore, this gap underscores the need for tailored physical activity recommendations that help patients achieve higher overall physical activity levels associated with better HR-QoL, while ensuring safety and adherence in real-world diabetes management.

In the adjusted analysis, HINT-8 utility index values were higher in the moderate and high physical activity groups than in the low physical activity group, although only the association in the high physical activity group reached statistical significance. This finding suggests that for adults with diabetes, simply meeting the minimum physical activity recommendations, which correspond to our moderate level, may not be sufficient to elicit discernible improvements in perceived health status [31]. Instead, our results indicate that reaching a high physical activity level may be more closely associated with achieving statistically significant HR-QoL gains. Although this 0.024-unit difference falls slightly below the commonly cited 0.03 minimal clinically important difference (MCID) established for the EQ-5D, the clinical significance should be weighed against the nature of the intervention. In contrast to pharmacological or invasive treatments, physical activity represents a low-cost and low-risk behavioral intervention, where even a 0.024-unit increment can be considered a meaningful gain that enhances patient self-efficacy and reduces the societal costs of lifelong diabetes management [36]. Furthermore, it is important to note that MCID values are not absolute; they exhibit substantial heterogeneity, ranging from 0.03 to 0.52, depending on the study population and the calculation method used [36, 37]. Given that a disease-specific MCID for the HINT-8 has not yet been established, our findings suggest that achieving a high level of activity may offer a worthwhile improvement in perceived well-being that outweighs the effort required for the behavior change.

In subgroup analyses, positive associations between physical activity level and the HINT-8 utility index were more apparent for high physical activity levels in several strata. Significant associations were observed among participants with a college education or higher, those in white-collar or inoccupation groups, and those with chronic diseases. Higher socio-economic resources, such as advanced education and professional occupation, are often linked to greater health literacy and self-efficacy, which may support sustained engagement in health-promoting behaviors and, in turn, better HR-QoL [38, 39]. Notably, no significant associations were found for blue- and pink-collar workers, even at high activity levels. This pattern is consistent with the “physical activity paradox,” suggesting that health implications of activity may differ by context [13]. While leisure-time physical activity is typically discretionary and allows recovery, occupational activity in manual jobs often entails sustained physical demands with limited recovery and high psychosocial stress, which may blunt potential HR-QoL gains [14, 40]. These findings suggest the potential value of occupation-sensitive strategies to support health literacy and feasible leisure-time activities with adequate recovery for adults with diabetes in physically demanding jobs. However, these occupational subgroup findings should be interpreted with caution. The pink-collar group was relatively small, and none of the formal interaction tests were statistically significant; therefore, the null findings may reflect limited statistical power rather than true biological differences and should not be interpreted as definitive evidence that the association differs by occupational group.

Importantly, the positive association between physical activity and the HINT-8 utility index was particularly pronounced in the cardiometabolic multimorbidity subgroup, which consisted of participants with diabetes who also had hypertension or dyslipidemia. This pattern may reflect the multi-system benefits of exercising, which has been described as a physiological “polypill,” particularly in individuals with a higher cardiometabolic burden [41]. Given the higher baseline vascular dysfunction and inflammation observed in these individuals, achieving a high physical activity level, representing a substantial total dose, may confer greater marginal improvements in vascular and cardiometabolic function, translating into larger gains in perceived well-being [39, 42]. These findings suggest that for adults with coexisting cardiometabolic conditions, a high level of physical activity could be an especially effective strategy for optimizing perceived health status and HR-QoL beyond conventional clinical targets.

This study had several limitations. First, the cross-sectional design precludes causal inference, and reverse causality remains possible. In addition, physical activity was assessed by self-report and may therefore be subject to recall and social desirability bias. Because both physical activity and HR-QoL were based on participant-reported data, common method bias cannot be excluded. Second, although we adjusted for a range of sociodemographic and health-related factors, residual confounding remains possible because several relevant behavioral and clinical variables, including dietary behaviors, diabetes duration, and diabetes-related complications, were not fully accounted for [43, 44]. We were unable to distinguish type 1 from type 2 diabetes, and the pooling of diabetes types may have introduced clinical heterogeneity and obscured subtype-specific associations with HR-QoL. The association between physical activity levels and HR-QoL was broadly defined and may have included a heterogeneous mix of retired and other economically inactive individuals, potentially obscuring meaningful variations in physical activity and HR-QoL both within this group and across occupational strata. This should be considered when interpreting occupation-related findings. Third, because data from 2021 were collected during the COVID-19 pandemic, pandemic-related behavioral changes may have influenced both physical activity and HR-QoL in ways that were not fully captured by adjustment for the survey year alone. Finally, although this study involved multiple subgroup analyses without formal adjustment for multiple comparisons, formal interaction tests for each stratification variable revealed no statistically significant effect modification. Accordingly, these subgroup findings should be interpreted as exploratory and hypothesis-generating rather than definitive evidence, and replication in future prospective studies is warranted.

Despite these limitations, this study has several notable strengths. First, the use of nationally representative KNHANES data supports population-level inferences among adults with diabetes. Second, the application of the HINT-8 utility index may provide greater sensitivity to subtle, diabetes-relevant deficits by capturing symptom domains such as vitality, memory, and sleep, which are often less well captured by generic instruments [17]. Third, the use of the GPAQ enabled a comprehensive assessment of physical activity levels by integrating intensity, frequency, and duration into the total activity dose across work, transport, and leisure domains. This approach facilitated a nuanced interpretation of occupational contexts and patterns consistent with the ‘physical activity paradox’ [26, 40]. Finally, by examining patterns across key subgroups and performing formal interaction tests, this study demonstrated the consistency and robustness of the association between high physical activity levels and HR-QoL across various demographic and clinical strata, offering evidence to support the broad applicability of these findings in adults living with diabetes.

## Conclusions

Using nationally representative KNHANES-VIII 2019–2021 data, this study found that high physical activity levels were associated with better HR-QoL among Korean adults with diabetes. Although the moderate physical activity group showed a positive association, only the high physical activity group differed significantly from the low physical activity group in the adjusted analyses. Despite these findings, a substantial proportion of adults with diabetes have reported low levels of physical activity, indicating a persistent gap between clinical recommendations and real-world practice. Significant positive associations with high physical activity primarily observed among participants with a college education or higher, those in white-collar or economically inactive groups, and those with cardiometabolic multimorbidity, however, as formal interaction tests were not statistically significant, these subgroup findings should be interpreted as exploratory. Collectively, our findings highlight the need for tailored context-sensitive strategies that support higher overall physical activity levels among adults with diabetes.

## Supplementary Information

Below is the link to the electronic supplementary material.

Supplementary file1 (PDF 327 kb)

## Data Availability

The data that support the findings of this study are publicly available from the Korea National Health and Nutrition Examination Survey (KNHANES) website [https://knhanes.kdca.go.kr/knhanes/main.do]. An English version of the website is available at [https://knhanes.kdca.go.kr/knhanes/eng/main.do]. Access is open to researchers upon request to the Korea Disease Control and Prevention Agency (KDCA) in accordance with their data use policy.
