# Supplementary material for: Physical activity levels and HINT-8 health-related quality of life in Korean adults with diabetes: analysis of KNHANES 2019–2021
Source: Qual Life Res. 2026 May 3;35(6):147. doi: 10.1007/s11136-026-04265-1 (PMC13136208; doi:10.1007/s11136-026-04265-1)
Supplement: Supplementary file 1 — Supplementary file1 (PDF 327 kb) [file 11136_2026_4265_MOESM1_ESM.pdf]

Journal: *Quality of Life Research*

**Article Title: Physical Activity Levels and HINT-8 Health-Related Quality of Life in Korean Adults with Diabetes: Analysis of KNHANES 2019–2021**

Sung Hoon Jeong[1,2,3±], You-Jung Choi[4±], Gain Shin[1,5], Ja-Ho Leigh[1,5,6*]

***Corresponding author**:

Ja-Ho Leigh, MD

Department of Rehabilitation Medicine, Seoul National University Hospital, 101 Daehak-ro, Jongno-gu, Seoul 03080, Republic of Korea

Email: jaho.leigh@gmail.com

**Supplementary Material**

**Table S1. Total physical activity levels**

| Total physical activity levels | Physical activity cut-off value |
|---|---|
| High | • At least 3 days of vigorous-intensity activity achieving a minimum of 1,500 MET-minutes per week<br>or<br>• 7 or more days of any combination of walking, moderate-, or vigorous-intensity activities achieving a minimum of 3,000 MET-minutes per week |
| Moderate | • 3 or more days of vigorous-intensity activity of at least 20 minutes per day<br>or<br>• 5 or more days of moderate-intensity activity and/or walking of at least 30 minutes per day<br>or<br>• 5 or more days of any combination of walking, moderate-, or vigorous-intensity activities achieving a minimum of 600 MET-minutes per week |
| Low | • Not meeting the criteria for either the 'High' or 'Moderate' categories |

MET, metabolic equivalent task
